# Treatment Disparities, Heterogeneities, and Barriers to Access for Patients with Hormone Receptor-Positive, Human Epidermal Growth Factor Receptor 2-Negative Metastatic Breast Cancer: A National Survey from Brazil

**DOI:** 10.3390/curroncol32080471

**Published:** 2025-08-19

**Authors:** Heloisa Resende, Vinícius de Q. Aguiar, Nataline F. de A. Santos, João Vitor Siqueira Jardim, André Ornelas

**Affiliations:** 1 Centro Universitário de Volta Redonda (UniFOA), Volta Redonda 27240-560, Rio de Janeiro, Brazil; viniciusqa@hotmail.com (V.d.Q.A.); nataline.freitas@yahoo.com.br (N.F.d.A.S.); jvsiqueira78@hotmail.com (J.V.S.J.); 2 Instituto Projeto CURA, São Paulo 05507-020, São Paulo, Brazil; 3 Novartis Brazil, São Paulo 04706-900, São Paulo, Brazil

**Keywords:** metastatic breast cancer, public health system, oncological assistance, medicines

## Abstract

Breast cancer (BC) is the most common malignancy among Brazilian women, with a high percentage of the cases diagnosed at advanced or metastatic stages (mBC). In Brazil, where 75% of the population depends on the resource-limited public health system (SUS), mBC poses significant treatment challenges and disparities. To characterize this scenario, we conducted an online survey assessing treatment strategies available for HER2-negative, hormone receptor (HR)-positive mBC across public and private health systems. The 48-question survey addressed topics such as waiting time (WT) from oncology unit entry to treatment initiation, availability of oncologic medications, and access to palliative and multidisciplinary care teams. Between 2 August 2022 and 30 September 2022, a total of 180 oncologists were invited, and 150 met the inclusion criteria. The median WT for surgery was 60 days in the SUS versus 30 days in the private sector (*p* < 0.0001), and for chemotherapy, 30 days in the SUS versus 15 days privately (*p* < 0.0001). Endocrine therapy was the preferred first-line treatment in the SUS (83.3%), while fulvestrant was available to only 48% of respondents. Additionally, specialized palliative care teams were available according to 66% of SUS respondents compared with 82% in the private system (*p* = 0.001). These findings underscore persistent disparities in mBC treatment, likely driven by limited governmental health investment.

## Simple Summary

Breast cancer is the most common cancer in Brazilian women, and many cases are diagnosed at advanced stages. Access to treatment can vary depending on whether patients are treated in the public or private health system. In this study, we surveyed oncologists across Brazil to understand how these differences impact the care of patients with a specific type of advanced breast cancer. We found that patients treated in the public system often experience longer waiting times to start treatment and have limited access to certain medications and palliative care services. Differences were also seen between regions of the country, highlighting further inequalities. These findings show that public investment and structural improvements in Brazil’s health system are needed to ensure that all patients receive timely and equitable cancer care. The results can inform policies to reduce disparities and improve treatment access for people with breast cancer in Brazil.

## 1. Introduction

Breast cancer (BC) is the most common neoplasm in women worldwide, accounting for 11.7% of all cancer cases [1]. In Brazil, an estimated 73,610 new cases are reported each year, according to the Instituto Nacional do Câncer (INCA) [2]. Additionally, a high percentage of BC cases are diagnosed at advanced stages, representing the leading cause of cancer-related death in women [3]. In addition to late diagnosis, other challenges, such as delays in starting treatment and the slow incorporation of new medicines into the public health system, should be addressed to improve outcomes [4,5,6]. Brazil and other low-middle-income countries (LMICs) have a significant percentage of their population covered by the public health system; therefore, understanding this system is critical for comprehending the journey of BC patients [7,8].

The Brazilian government created its public health system (Sistema Único de Saúde—SUS), which has universal coverage and has received continuous improvements to reduce inequities [9]. Despite these efforts, disparities in access to healthcare, including oncological care, persist [10,11].

The SUS public health system with universal coverage in Brazil was established in 1990, and oncological assistance has been provided within its principles [9]. In the first decade, oncological care was provided in general hospitals and outpatient clinics, offering fragmented care, meaning that patients received treatments in different places, as hospitals and clinics were not dedicated to assisting cancer patients [12]. An important disadvantage of this model is the lack of a clear obligation for each care provider institution to assist patients during their clinical complications related to treatment and malignant disease.

Two ministerial ordinances published in 1998 are considered landmarks within the SUS regarding oncological care, as they established guidelines that continue to shape public oncology services today. The first ordinance (No. 3535) created the requirements to accredit hospitals as high complexity cancer care centers (Centros de Assistência de Alta Complexidade em Oncologia—CACONs) and criteria to estimate the necessity of hospitals to be credentialed according to epidemiological data [13]. As a result of this ordinance, oncological care was centralized and integrated within hospitals, with no further accreditation granted to new service providers outside hospital settings. Existing outpatient clinics were given deadlines to comply with the new regulations. The other ordinance (No. 3536) created codes for radiotherapy and chemotherapy procedures, which should be used within a subsystem of the SUS, the outpatient information system (Sistema de Informação Ambulatorial—SIA-SUS) [14]. These codes should be used for the authorization of chemotherapy and radiotherapy procedures (Autorização Procedimento de Alto Custo—APAC). These ordinances represent the beginning of public oncological assistance centralized in hospitals, with payments for individual procedures traceable by APAC codes. Such points have provided benefits for patients who have been assisted during their clinical complications at the same hospital where they have undergone surgery, chemotherapy, or radiotherapy, and administrative advantages, avoiding fraud.

Ministerial ordinances have been updated with complementary points, such as creating a Unit of Oncological Assistance (Unidade de Assitência de Alta Complexidade em Oncologia—UNACON), which permits the accreditation of small hospitals that assist more prevalent tumors in each region [15]. As a result of these strategies, public oncological assistance in Brazil is currently offered in both public and private hospitals certified by the government. Another landmark was the creation of the National Commission of Incorporation of New Technologies in the SUS (Comissão Nacional de Incoproração de Tecnologias—CONITEC) in 2011 [16]. CONITEC is a permanent commission advising the Ministry of Health regarding the incorporation, exclusion or alteration of new procedures, medicines, and therapeutic and diagnostic guidelines [16].

The implementation of the SUS has improved healthcare assistance; however, the Brazilian government has yet to adopt a clear public policy for oncological care, and the SUS has remained underfunded since its inception [17,18]. On the other hand, the private health system is regulated by a governmental supplementary health agency (Agência Nacional de Saúde Suplementar—ANS), which manages adjustments and establishes rules for private health insurance companies. ANS has advocated the incorporation of new therapies after their approval by the Brazilian Health Regulatory Agency (Agência Nacional de Vigilância Sanitária—ANVISA).

Given the historical context of structural differences between Brazil’s public and private healthcare systems, it is crucial to investigate how such disparities influence access to cancer care services. Furthermore, it is necessary to understand regional differences within SUS, which are critical elements driving disparities. To address these, we conducted a survey with Brazilian clinical oncologists working in both public and private healthcare sectors, focusing on identifying treatment disparities, heterogeneities, and the barriers faced by patients with human epidermal growth factor receptor 2 (HER2)-negative, hormone receptor (HR)-positive metastatic breast cancer (mBC) in the public healthcare system compared with the private sector and the same points within SUS, across five geographical regions.

## 2. Materials and Methods

We developed an online survey comprising 48 questions, structured into three sections: (1) demographic data, with seven questions; (2) SUS practice, with 26 questions about waiting time (WT) from the registration date at the oncological care unit to first treatment, availability of systemic agents (chemotherapy and endocrine therapy), and some questions involving supportive care; and (3) private health system practice, with 15 questions about WT from the registration date at the oncological care unit to first treatment, options for treatment in mBC, and supportive care questions (Supplementary Material). The questionnaire was preliminarily administered to six clinical oncologists to assess the content validity, comprehensibility, and acceptability of the survey items. The final questionnaire was programmed and distributed, and raw data were managed by Pesquisa e Dados, an independent Brazilian market-research firm, under the investigator’s supervision.

According to the 2018 Brazilian Medical Census, approximately 3583 clinical oncologists were identified across the country [19]. Their geographic distribution was as follows: North (6.3%), Center-West (9.5%), Northeast (18.3%), South (21.5%), and Southeast (46.5%). Given the unequal distribution of oncologists across regions—each with distinct healthcare structures and clinical realities—the sampling strategy was adjusted to ensure proportional representation. This approach aimed to minimize potential distortions in the results caused by regional overrepresentation or underrepresentation of oncologists. Based on these considerations, a total sample size of 150 participants was calculated to estimate the expected proportions with an absolute precision of 7.8% and a 95% confidence interval. Clinical oncologists were eligible if they had assisted patients with BC in both public and private healthcare systems between January 2018 and January 2020 and consented to data collection. Data were collected from 2 August 2022 to 30 September 2022, but the questions referred to the period before COVID-19 (January 2018 to January 2020), and it was clearly specified.

A total of 180 clinical oncologists were interviewed; however, 30 of them were excluded from the data analysis: 6 participants from the pilot phase and 24 who did not meet the inclusion criteria (they worked exclusively in private or exclusively in the public health system). The questionnaire was administered following the oncologists’ consent and agreement to participate in the survey. The oncologists who participated in the survey were compensated by *Pesquisa e Dados*. This compensation was intended to acknowledge their time and effort and was not based on the content of their responses. To ensure that compensation did not influence the objectivity or integrity of the data collected, participants were informed in advance that remuneration was fixed and unrelated to their answers, and all responses were anonymized prior to analysis.

This study was reviewed and approved by the Fundação Oswaldo Aranha—UNIFOA Ethics Committee under the approval number 58853722.5.0000.5237.

### Statistical Analysis

The survey consisted of multiple-choice questions, and the respondents were not required to answer all of them. Continuous variables were measured using median and range, whereas categorical variables were expressed as proportions. The Z-test was used to compare the proportions of the two independent groups. Additionally, a two-sided chi-square test for independence was used to compare the observed and expected frequencies in the contingency tables. A 5% level of statistical significance was used for both tests. Exploratory statistical techniques were used to analyze the data and enhance the visualization of general characteristics. Data are presented in frequency tables with absolute frequencies and corresponding percentages. Comparisons of WT between groups were performed using the Mann–Whitney U test, as WT data were not normally distributed. Analyses were performed using the SPSS statistical software (version 25.0).

## 3. Results

**Figure 1:**
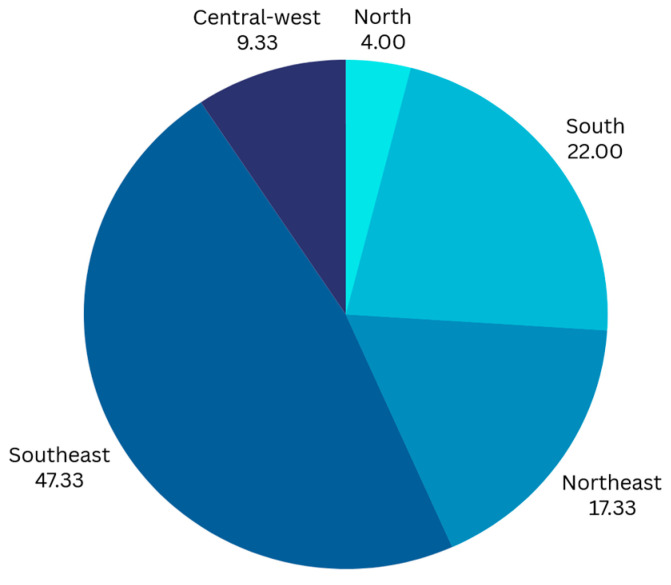
Distribution (%) of respondents in Brazilian geographic regions.

A total of 150 eligible oncologists completed the survey. The majority were based in the Southeast region (*n* = 71, 47.3%), followed by the South (*n* = 33, 22.0%), Northeast (*n* = 26, 17.3%), Central-West (*n* = 14, 9.3%), and North (*n* = 6, 4.0%) (Figure 1). The median time working as a clinical oncologist was 8.0 years (range: 1.0–30.0 years), with a median of 50.0% (range: 10.0–95.0%) of time dedicated to the public health system (SUS). Respondents reported a median of 90.0% (range: 30.0–100.0%) of their professional time devoted to direct patient care. Most respondents (*n* = 147, 98.0%) reported being directly responsible for treatment decisions (Table 1).

### 3.1. Waiting Time to Initial Treatment

**Figure 2:**
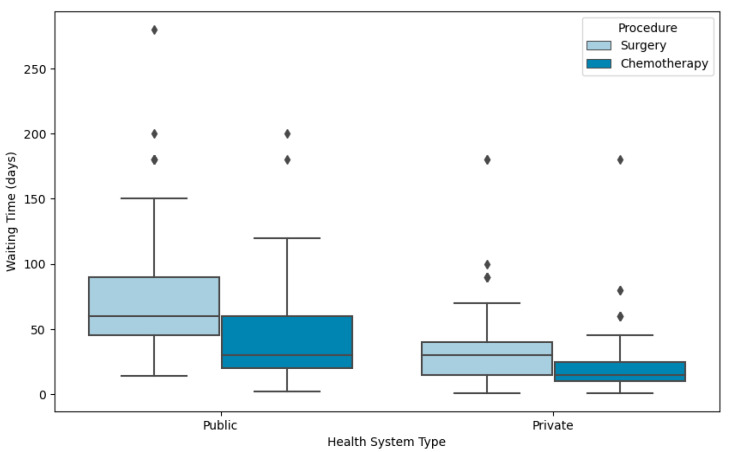
Waiting time (days) for initial treatment (surgery or chemotherapy) in the public and private health systems.

Among patients with non-metastatic disease and an indication for surgery as initial treatment, the median WT from diagnosis to surgery was 60 days (range: 14–280) in the public setting, compared to 30 days (range: 1–180) in the private sector (*p* < 0.0001) (Figure 2). Regional variation in WT was observed in the public system: Southeast, South, and Northeast had a median of 60 days; Central-West, 90 days; and North, 150 days. In contrast, private sector WTs were shorter and more homogeneous, with a median time of 30 days in the Southeast, North and Northeast; 21 days in the Central-West region; and 20 days in the South region.

When chemotherapy was the first modality of treatment (for either metastatic or non-metastatic disease), the median WT from diagnosis to chemotherapy initiation was 30 days (range: 2–200) in the public system (SUS) and 15 days (range: 1–180) in the private system (*p* < 0.0001). Within the SUS, median WT varied across regions: 25.0 days in the South, 32.5 days in the Northeast, 40.0 days in the Southeast, 47.5 days in the North, and 50.0 days in the Central-West.

### 3.2. Use and Availability of Treatments

In the public system setting, endocrine therapy was the preferred first-line treatment for metastatic disease, reported by 83.3% (*n* = 125) of respondents. In contrast, chemotherapy was chosen by 16.7% (*n* = 25).

**Figure 3:**
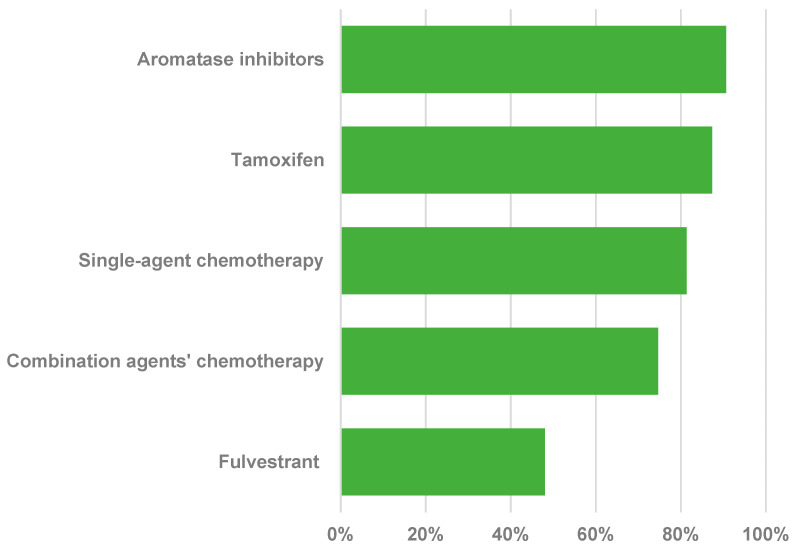
Percentage of agents available in metastatic setting in the public health system.

**Figure 4:**
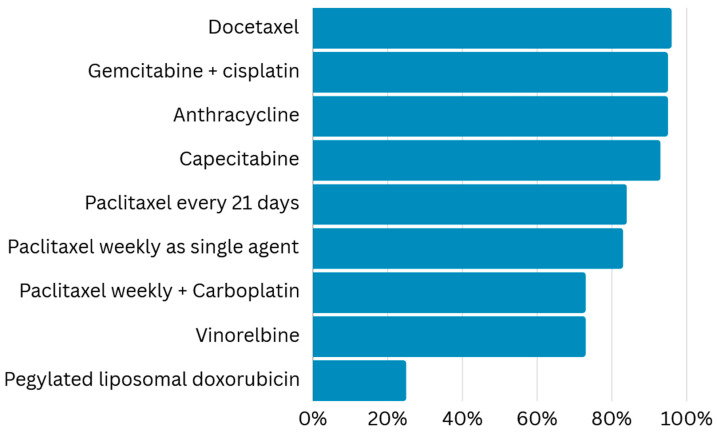
Chemotherapy agents available in the public health system.

Regarding treatment availability in the first-line setting, except in the context of visceral crisis, aromatase inhibitors (AIs) were available for 136 (90.6%) respondents, tamoxifen for 131 (87.3%), single-agent chemotherapy for 122 (81.3%), and combination chemotherapy agents for 112 (74.6%). Fulvestrant was available for only 72 (48.0%) of oncologists (Figure 3). Regarding chemotherapy agents, anthracycline, capecitabine, docetaxel, and gemcitabine plus cisplatin were available for more than 140 oncologists (range 140–144, 96.3%). Weekly paclitaxel alone or in combination with platinum was available for 124 (82.6%) and 109 (72.6%), respectively. Vinorelbine was available for 109 respondents (72.6%). Pegylated liposomal doxorubicin (PLD) was available for only 38 (25.3%) oncologists (Figure 4). With respect to the prescription of osteolysis inhibitors in patients with bone metastasis, zoledronic acid was most prevalent every 12 weeks (*n* = 75, 50.0%), followed by the same medicine every 3 or 4 weeks (*n* = 51, 34.0%), and pamidronate every 3 or 4 weeks (*n* = 23, 15.3%).

In the private sector, in the first-line setting, most surveyed oncologists (*n* = 137, 91.3%) preferred endocrine therapy plus cyclin-dependent kinase (CDK) 4/6 inhibitors, and most of them (*n* = 117, 78.0%) considered it easy to access CDK4/6 inhibitors through health insurance coverage.

### 3.3. Operational Aspects of Oncology Care Delivery in the Public System

In the public setting, when changes in chemotherapy protocols were necessary (e.g., due to disease progression), the median WT for implementation was 15.0 days (range: 0–400). Regarding vascular access, 131 of 150 oncologists (87.3%) reported routinely requiring a port-a-cath, with a median WT of 40.0 days (range: 7–120).

Only 46 respondents (30.7%) indicated that supportive medications were dispensed directly at the oncology care unit. A mere 22.0% (*n* = 33) agreed or strongly agreed that there was a service available to issue death certificates for patients who died at home. Concerning the number of treatment lines available, 108 oncologists (71.7%) agreed or strongly agreed that patients could receive multiple lines of therapy as needed, depending on SUS drug availability.

### 3.4. Availability of Multidisciplinary and Palliative Care

Regarding multidisciplinary support, 77.3% of respondents agreed or strongly agreed that such teams were available in the SUS, versus 87.3% in the private system (*p* = 0.022). Access to specialized palliative care services was reported by 66.0% of oncologists in the SUS and 82.0% in the private sector (*p* = 0.001).

Among patients receiving exclusive palliative care, 25.3% in the SUS and 46.7% in the private sector had regular oncologist consultations every 30 days. Irregular intervals were reported by 22.7% in the SUS versus 16.7% in the private system (*p* = 0.045).

## 4. Discussion

Brazil is a continental country with many social and demographic heterogeneities reflected in oncological care. Within the public health system, there are significant differences in the medicines available for treating mBC. For example, PLD and fulvestrant were available for one-quarter and two-quarters of respondents, respectively. We also demonstrated a difference in the median WT for the treatment reported by oncologists within the SUS. These findings are unexpected within the SUS, as the treatment is funded and regulated by the federal government, which is unified across the country. We also demonstrated inequities between private and public systems regarding WT for first-time treatment, palliative, and multidisciplinary team access.

The surveyed oncologists worked in public and private health systems, which allowed us to compare oncological care between the two settings within the same region at a few points. The five Brazilian regions were represented proportionally to the number of oncologists in each region to guarantee representativity; however, it was not possible to establish comparisons among the regions owing to the small sample size.

Regarding non-metastatic BC surgery, the median WT in the public health system was 60 days, which was double the duration observed in the private health system (30 days). Particularly concerning were the prolonged median WTs reported in the North (150 days) and in the Central-West (90 days). We believe that these regional disparities in WT are likely multifactorial, reflecting underlying inequities in healthcare infrastructure, availability of specialized cancer services, and resource allocation across different regions of Brazil. Although the WT reported in this study reflects only the interval from patient registration at the oncological care unit to surgery—and does not include the time from pathological diagnosis to referral and registration, as defined by legal benchmarks such as Law No. 12.732/12, which establishes a 60-day timeframe starting from the date of the pathological report release—our results capture an integral part of the overall treatment timeline. Therefore, it is reasonable to expect that the total WT likely exceeds the legally established 60-day target [20]. Previous studies have also reported substantial delays in Brazil from self-detected abnormalities to diagnosis [21,22,23]. In this context, the 30-day WT reported for the private health system is close to the 21-day timeframe stipulated by the ANS for private care [24]. Such disparities may also be influenced by the regulatory frameworks governing surgical procedures in Brazil’s healthcare systems. Surgical procedures in the public health system are regulated by ministerial ordinances, whereas in the private system, they are regulated by the ANS. Ordinances 170 and 171, published in 1993, created 119 surgical procedure codes, with updates in 2012 and 2022, resulting in approximately 400 procedures [25,26,27]. This encompasses all possible surgical procedures within the SUS; however, there is no regular governmental policy regarding reimbursement adjustments, which may further affect service provision and access.

The median WT for chemotherapy was 30 days in the public health system and 15 days in the private health system. Hospital remuneration and regulatory rules for chemotherapy differ significantly between public and private systems. In the private sector, chemotherapy is regulated by the ANS and new medications are incorporated as soon as they receive approval by ANVISA. In the public system, the qualified hospital, after patient admission, requires chemotherapy via APAC emission, which encompasses 42 fields, including patient identification, International Classification of Disease (ICD), other tumor characteristics, a short description of chemo or endocrine protocols, and the procedure code [28]. The information required inside APAC is part of the data that will feed the information systems of the Ministry of Health. The code for the main procedure is linked to the amount reimbursed for the required procedure (chemotherapy or endocrine therapy), which can also be consulted on an electronic site [28]. The local manager (representing the municipal sphere) is responsible for evaluating and approving this document, thereby ensuring the corresponding payment.

There is no formal or updated protocol recommended by the Brazilian government regarding chemotherapy and endocrine therapy. The Brazilian government has a technical manual containing instructions to guide the solicitations and authorizations of oncological procedures; however, it is not a treatment protocol [29]. Some medical societies and national leaders have independent publications; therefore, in the real world, what regulates the systemic treatment protocol in the public health system is the protocol cost, restricted by the reimbursement received with APAC [30]. This means that if a certain code is related to chemotherapy, for example, it will encompass medications whose costs are covered by the amount reimbursed by the APAC.

The combination of CDK 4/6 inhibitor plus endocrine therapy is the first-line option for patients with HR-positive, HER2 negative mBC and the first option recognized by more than 90% of the interviewed oncologists; however, it is not available in the public system because, as of May 2025, the Brazilian Ministry of Health has not allocated funds for a specific APAC for CDK 4/6 inhibitor [31]. Although it has received a favorable note from CONITEC regarding its incorporation in the SUS, it remains unavailable [4,32]. CONITEC is a collegiate body that has a consultative role; however, after notification, the incorporation of new procedures is not immediate, requiring strategies for granting funding and monitoring incorporation.

According to the opinion of the Brazilian Clinical Oncological Society (SBOC), the current model creates a scenario with three levels of hospitals: (1) hospitals that offer assistance superior to what the protocol/public system protocol; (2) hospitals that offer what the government recommends; and (3) hospitals that provide less than what is recommended. Unfortunately, this last category represents most of the care providers in Brazil, reinforcing the heterogeneity and inequities within the public health system [33].

Another way that oncological medicines are provided by the Brazilian government, representing rare situations, is through the centralized purchasing of medicines, such as trastuzumab and pertuzumab, by the Ministry of Health [34]. This mechanism of funding oncological medicines is based on negotiations between pharmaceutical companies and the government, which leads to lower prices, rather than negotiations between pharmaceutical companies and health care service providers [29,35,36,37]. This strategy may be strengthened, enabling faster incorporation of new drugs.

Notably, biologic therapies were not included in our availability questions because, for selected oncology biologics supplied through centralized purchasing and federal distribution, availability does not depend on hospital-level budgets or APAC coding—the inequity mechanism under investigation in this survey. Including these agents would not meaningfully reflect the between-hospital disparities our instrument was designed to capture.

Importantly, at the time of the study, all treatment options assessed were considered part of the standard of care for breast cancer management in Brazil, according to national guidelines. Regarding available protocols, nearly 90% of the surveyed oncologists reported having access to tamoxifen and AIs, around 80% reported having access to chemotherapy (either as a single agent or in combination), but only 48% reported having access to fulvestrant. The next question specifically evaluated which chemotherapeutic agents (docetaxel, paclitaxel, capecitabine, vinorelbine, gemcitabine plus cisplatin, or PLD) were available. Most oncologists reported having docetaxel, doxorubicin, capecitabine, and gemcitabine plus cisplatin; close to 70% reported having vinorelbine and weekly paclitaxel plus carboplatin, but only one-quarter reported having access to PLD. The low availability of fulvestrant and PLD might reflect the higher costs related to these medicines, creating or reinforcing disparities within the SUS [4,38]. The Brazilian government published a new ordinance in March 2022, aiming to regulate chemotherapy protocols, making protocol information in field number 32 obligatory and correlating it with protocols evaluable within the APAC system (there were no registered protocols before this ordinance) [39]. However, the effects of this ordinance were not evaluated.

Both surgical and clinical procedures within the SUS are regulated by ministerial ordinances that have received several updates, including new procedures that are necessary. However, there is no clear policy for adjusting the values paid by the government, which hinders improvement in oncological care. In other countries across Latin America and the Caribbean, a similar scenario has been observed regarding the disparities between public and private healthcare systems. These disparities are likely exacerbated by limited government investment, leading to the absence of clear cancer control policies. As a result, there is a high prevalence of late-stage diagnoses, delays in the incorporation of new medications, and consequently, higher mortality rates compared with rates in very high-income countries (HICs) [40,41]. Although Brazil spends a proportion of its gross domestic product (GDP) higher than the average GDP of the Organization for Economic Cooperation and Development (OECD) countries, 60% of this expenditure comes from the private sector [42,43]. In 2011, the total health expenditure in Brazil was 7.8% of its GDP, with only 3.6% (44.9% of 7.8%) provided by the government and 4.2% (55.1% of 7.8%) provided by private health insurance. Despite the CONITEC creation in 2011, which is supposed to increase the incorporation of new medicines and procedures, 8 years later, the global health expenditure in 2019 was 9.6% of the Brazilian GDP, but only 3.9% (40.6% of 9.6%) was provided by the government, which represents 4.3% less than what was allocated in 2011 (44.9% of the GDP provided by government in 2011 versus 40.6% of GDP provided by Government in 2019) [44,45]. Health expenditure by the public sector in Brazil in 2019 (40.6% of the total GDP) was lower than the average of the largest economies (64.1%) and even lower than the public health expenditure average in Latin American countries (56.7%) [45]. Low health investment by the Brazilian government is one factor that might explain the difference in cancer patients’ outcomes between LMICs and high-income countries (HICs) [18,46].

Although this study provides critical insights into the landscape of breast cancer treatment in Brazil, its limitations must be carefully considered when interpreting the findings. First, the methodology based on an online survey is subject to inherent biases, such as recall bias, given that data collection occurred between August and September 2022, but the questions referred to the period from January 2018 to January 2020. Additionally, the total sample size, although proportionally representative of the distribution of oncologists in the country, lacks the statistical power to allow for robust comparisons between geographic regions. Consequently, data from regions with a smaller number of respondents should be interpreted with caution and viewed as exploratory. Another crucial point is the temporal context, as the data reflect a pre-COVID-19 pandemic scenario, not capturing the subsequent impact that the health crisis may have generated on disparities in oncological care.

Despite these limitations, the study successfully describes the differences in available medicines within the SUS and disparities between the SUS and private systems, highlighting the need for governmental strategies to improve cancer care in the country.

## Figures and Tables

**Table 1:** Respondents’ baseline characteristics.

Characteristic	Measure (*N* = 150)
Years working as oncologist—median (range)	8 (1.–30.0)
Percentage of time dedicated to SUS *—median (range)	50 (10.00–95.00)
Percentage of time dedicated to clinical practice—median (range)	90 (30.00–100.00)
Responsible for treatment decision—*n* (%)	147 (98.0%)

* **SUS**: Sistema Único de Saúde.

## Data Availability

Study protocol and data, including study-level data (analysis datasets), as well as other information (e.g., study reports or analysis plans), are available upon request to the investigators. These documents can be requested by any qualified researchers who engage in rigorous, independent, scientific research and will be provided following review and approval of a request. Requests can be submitted to the corresponding author at any time after the publication of this manuscript.

## Supplementary Materials

The following supporting information can be downloaded at https://www.mdpi.com/article/10.3390/curroncol32080471/s1, the complete survey questionnaire.

## Abbreviations

The following abbreviations are used in this manuscript:

BC	Breast Cancer
mBC	Metastatic Breast Cancer
SUS	Sistema Único de Saúde (Unified Health System, Brazil’s public health system)
HER2	Human Epidermal Growth Factor Receptor 2
HR	Hormone Receptor
WT	Waiting Time
INCA	Instituto Nacional do Câncer (National Cancer Institute, Brazil)
LMICs	Low- and Middle-Income Countries
CACONs	Centros de Assistência de Alta Complexidade em Oncologia (High Complexity Oncology Care Centers)
UNACON	Unidade de Assistência de Alta Complexidade em Oncologia (High Complexity Oncology Care Unit)
SIA-SUS	Sistema de Informação Ambulatorial do SUS (Outpatient Information System of SUS)
APAC	Autorização Procedimento de Alto Custo (Authorization for High-Cost Procedures)
CONITEC	Comissão Nacional de Incorporação de Tecnologias (National Commission for the Incorporation of Technologies)
ANS	Agência Nacional de Saúde Suplementar (National Supplementary Health Agency)
ANVISA	Agência Nacional de Vigilância Sanitária (National Health Surveillance Agency)
CDK 4/6	Cyclin-Dependent Kinase 4/6
AIs	Aromatase Inhibitors
PLD	Pegylated Liposomal Doxorubicin
GDP	Gross Domestic Product
OECD	Organisation for Economic Co-operation and Development
SBOC	Sociedade Brasileira de Oncologia Clínica (Brazilian Society of Clinical Oncology)
WHO	World Health Organization
ICD	International Classification of Diseases
HICs	High-Income Countries
