# Supplementary material for: Treatment Disparities, Heterogeneities, and Barriers to Access for Patients with Hormone Receptor-Positive, Human Epidermal Growth Factor Receptor 2-Negative Metastatic Breast Cancer: A National Survey from Brazil
Source: Curr Oncol. 2025 Aug 19;32(8):471. doi: 10.3390/curroncol32080471 (PMC12384988; doi:10.3390/curroncol32080471)
Supplement: Supplementary file 1 [file curroncol-32-00471-s001.zip › curroncol-3761339-supplementary.pdf]

IDENTIFICATION

1. Considering the public and private health systems, how long have you been working as an oncologist in Brazil?

(Provide your answer in years).

___________________ years

2. In Brazil, in which geographical region do you work as an oncologist?

(Choose as many options as necessary)

[ ] Southeast

[ ] South

[ ] North

[ ] Northeast

[ ] Central-West (including the Distrito Federal)

3. Considering the period between January 2018 and January 2020 (before the COVID-19 pandemic), evaluate the following statement.

I have worked as an oncologist at an Unidade de Assistência de Alta Complexidade (UNACON).

[ ] Strongly agree

[ ] Agree

[ ] Neither agree nor disagree

[ ] Disagree

[ ] Strongly disagree

4. Considering the period between January 2018 and January 2020 (before the COVID-19 pandemic), evaluate the following statement.

I have worked as an oncologist at a Centro de Assistência de Alta Complexidade em Oncologia (CACON).

[ ] Strongly agree

[ ] Agree

[ ] Neither agree nor disagree

[ ] Disagree

[ ] Strongly disagree

5. As oncologists can have various professional activities in addition to direct patient care (e.g., teaching, research, administrative and political activities), this question is about the percentage of time you dedicated exclusively to patient care.

Considering the period from January 2018 to January 2020 (before the COVID-19 pandemic), what was the percentage of your time as an oncologist dedicated exclusively to patient care?

(Provide your best guess in days; any number from 0 to 100).

__________________ %

6. Evaluate the following statement:

In the period between January 2018 and January 2020 (before the COVID-19 pandemic), I worked as an oncologist and was directly responsible for treating cancer patients in Brazil.

[ ] Strongly agree

[ ] Agree

[ ] Neither agree nor disagree

[ ] Disagree

[ ] Strongly disagree

7. This question is about the proportion of time (per week) you dedicate to the Sistema Único de Saúde (SUS) as an oncologist.

Considering the period from January 2018 to January 2020 (before COVID-19), what was the percentage of your working time as an oncologist dedicated to patient care in SUS?

(Provide your best guess in percentage; any number from 0 to 100).

__________________ %

SUS

1. Now the question is about the "typical" time from the diagnosis of metastatic breast cancer to the surgical treatment at the public institution you work for.

Consider you have a female patient who has been recently diagnosed with metastatic breast cancer. The patient is 54 years old and has never been treated for breast cancer before. Based on the careful evaluation of her clinical and pathological characteristics, it was concluded that she has an indication for surgical treatment. Considering the period between January 2018 and January 2020 (before the COVID-19 pandemic), what would be the waiting time (in days) from diagnosis to surgical treatment for that patient at the public institution you work for?

(Provide your best guess in days; any number from 1 to 1,000).

__________________ days

2. Consider a patient with metastatic breast cancer when she was first diagnosed with breast cancer ("de novo metastatic"). The patient has an indication to start treatment at the clinical oncology sector via SUS.

Considering the period between January 2018 and January 2020 (before the COVID-19 pandemic), what would be the waiting time (in days) from diagnosis to clinical treatment for that patient at the public institution you work for?

(Provide your best guess in days; any number from 1 to 1,000).

__________________ days

3. Now the question is about the availability of hormone therapy (also termed endocrine therapy) to treat the pre-menopause breast cancer patients at the public institution you work for.

Assume you have a Premenopausal, high-risk patient with hormone-positive/HER2- breast cancer, who has already been submitted to surgical treatment (stage I to III). Considering the period between January 2018 and January 2020 (before the COVID-19 pandemic), check all protocols below that were available via the Sistema Único da Saúde (SUS) to treat that patients at the public institution you work for in the adjuvant setting:

(Check as many as necessary)

[ ] Tamoxifen alone

[ ] Tamoxifen plus luteinizing hormone-releasing hormone agonists

[ ] Tamoxifen plus oophorectomy

[ ] Aromatase inhibitor plus luteinizing hormone-releasing hormone agonists

[ ] Aromatase inhibitor plus oophorectomy

[ ] Other. Please, specify: ______________________________

4. Based on your experience as an oncologist in SUS from January 2018 to January 2020 (before the COVID-19 pandemic), what was the most frequent treatment you prescribed for your patients with the following characteristics?

- High-risk Breast cancer patients treated via SUS
- Premenopausal
- Surgically treated via SUS
- hormone-positive/HER2-

(Specify here)

____

5. The focus of this question is on how you treat your patients at the public service you work for.

Consider you have a 59-year old patient with hormone receptor-positive/HER2-negative metastatic breast cancer treated via the Sistema Único de Saúde (SUS). She has no history of visceral crisis and has received one line of adjuvant hormone therapy. Considering the period between January 2018 and January 2020 (prior to the COVID-19 pandemic), which of the following was the next most appropriate treatment (first treatment after diagnosis of metastasis) in her management in SUS?

[ ] Hormone therapy

[ ] Oral single agent chemotherapy

[ ] Venous single-agent chemotherapy

[ ] Oral and/or venous chemotherapy with a combination of agents

[ ] Other. Please, specify: ______________________________

6. Now the question is about the availability of treatments in the Sistema Único de Saúde (SUS) to manage metastatic breast cancer patients. More specifically, the population of interest is women with hormone receptor-positive/HER2-negative metastatic breast cancer and no history of visceral crisis. Based on your experience as an oncologist, which of the following regimens were available as first-line treatments to treat your patients with those characteristics in SUS from January 2018 to January 2020 (before the COVID-19 pandemic)?

(Choose as many options as necessary)

[ ] Hormone therapy with aromatase inhibitors

[ ] Tamoxifen

[ ] Fulvestrant

[ ] Single-agent chemotherapy

[ ] Chemotherapy as a combination of agents

[ ] Other. Please, specify: ______________________________

7. Evaluate the following sentence:

“In SUS, considering the period between January 2018 and January 2020 (before the COVID-19 pandemic), treatment with fulvestrant was available for managing my breast cancer patients who have already received tamoxifen and an aromatase inhibitor but progressed to metastatic disease without visceral crisis:

[ ] Strongly agree

[ ] Agree

[ ] Neither agree nor disagree

[ ] Disagree

[ ] Strongly disagree

(if Strongly agree or Agree)

7.b. Regarding the treatment with fulvestrant in SUS, check the regimen you have evaluablefor breast cancer patients who have already received tamoxifen and an aromatase inhibitor but progressed to metastatic disease without visceral crisis:

[ ] fulvestrant 250 mg every 28 days

[ ] fulvestrant 500 mg every 28 days

[ ] fulvestrant 250 mg with a dose on D14 in the first cycle

[ ] fulvestrant 500 mg with a dose on D14 in the first cycle

[ ] Other. Please, specify: ______________________________

8. This next question is about which chemotherapy agents are available in SUS – based on your experience as an oncologist in the public sector.

For the treatment of patients with hormone-positive/HER2- metastatic breast cancer, which of the following therapies were available between January 2018 and January 2020 (before the COVID-19 pandemic) in SUS?

[choose as many as necessary]

[ ] Docetaxel every 21 days

[ ] Weekly paclitaxel as a single agent

[ ] Paclitaxel every 21 days (as a single agent or in combination)

[ ] capecitabine

[ ] Oral navelbine

[ ] Gemcitabine and platinum

[ ] Weekly paclitaxel combined with platinum

[ ] anthracycline [ ] Other. Please, specify: ______________________________

9. The following question refers to the average time (in days) to receive authorization to change the treatment protocol of a cancer patient in SUS.

Consider a 59-year-old woman with metastatic breast cancer who has been treated in SUS with docetaxel. After assessment, you decided to change her medication to capecitabine due to disease progression. Based on your experience from January 2018 to January 2020 (before the COVID-19 pandemic), how long would it take (in days) to have the authorization to change the treatment protocol for that patient in SUS?

(Provide your best guess in days; any number from 1 to 1,000).

__________________ days

10. The next question is about the number of lines of treatment that your patients can receive when treated in SUS. Evaluate the following sentence:

"In SUS, there is no limitation regarding the number of lines of treatment that my hormone-positive/HER2- metastatic breast cancer patients undergoing palliative chemotherapy can receive. My patients can receive as many lines of treatment as needed."

[ ] Strongly agree

[ ] Agree

[ ] Neither agree nor disagree

[ ] Disagree

[ ] Strongly disagree

11. Between January 2018 and January 2020 (before the COVID-19 pandemic), did you prescribe totally implantable venous catheters for chemotherapy for your breast cancer patients treated via SUS?

[ ] Yes

[ ] No

[ ] I don't remember

[If yes]

11.b. You responded that you prescribed totally implantable venous catheters for chemotherapy for your breast cancer patients treated via SUS. Now the question is related to the easeness of access to fully implantable venous catheters for chemotherapy in SUS (how easy it is).

Between January 2018 and January 2020 (before the COVID-19 pandemic), access to a totally implantable venous catheter for chemotherapy for my breast cancer patients treated via SUS was easy.

[ ] Strongly agree

[ ] Agree

[ ] Neither agree nor disagree

[ ] Disagree

[ ] Strongly disagree

11.c The question now focuses on the amount of time (in days) required for patients to access totally implantable venous catheters for chemotherapy in SUS.

Based on your experience between January 2018 and January 2020 (before the COVID-19 pandemic), how many days did it take for your breast cancer patients to receive the totally implantable venous catheter via SUS?

(Provide your best guess in days; any number from 1 to 1,000).

_________ days

12. This next question focuses on the frequency with which medical consultations were scheduled in SUS for your patients who receive chemotherapy. Consider your patients with metastatic breast cancer who received chemotherapy via SUS between January 2018 and January 2020 (before the COVID-19 pandemic).

Choose the option below that best describes the interval between their medical consultations.

[ ] Every 7 days

[ ] Every 15 days

[ ] Every month

[ ] Every two months

[ ] More than two months

13. This question asks about the healthcare pathway (journey) of your patients. More specifically, whether or not patients received radiotherapy at the same service they were being treated.

Consider your metastatic breast cancer patients who have received chemotherapy via SUS. The patients had an indication of radiotherapy to alleviate pain and prevent bleeding (hemorrhages).

Considering the period between January 2018 and January 2020 (before the COVID-19 pandemic) and your experience in SUS, did the service offer radiotherapy at the same medical service that they were treated?

[ ] Yes

[ ] No

[ ] I don't know

14. Considering the treatment of your breast cancer patients with bone metastases treated via SUS, which osteolysis inhibitors do you prescribe?

[ ] Pamidronate every 21 or 28 days

[ ] Zoledronic acid every 21 or 28 days

[ ] Zoledronic acid every 12 weeks

[ ] Other. Specify ________________________________

14.b. Also in relation to the prescription of osteolysis inhibitors in SUS, evaluate the following statement:

I can choose freely between pamidronate and zoledronic acid and define their doses/dosage according to my patients' needs.

[ ] Strongly agree

[ ] Agree

[ ] Neither agree nor disagree

[ ] Disagree

[ ] Strongly disagree

15. Evaluate the following sentence:

Between January 2018 and January 2020 (before the COVID-19 pandemic), in SUS, I had support from a multidisciplinary cancer team to manage my metastatic breast cancer patients at the public service I work for.

[ ] Strongly agree

[ ] Agree

[ ] Neither agree nor disagree

[ ] Disagree

[ ] Strongly disagree

(If Strongly agree or Agree)

15.b. This question is regarding the characteristics of the multidisciplinary cancer team that supported the management of your metastatic breast cancer via SUS. Select all professional categories that were part of the multidisciplinary cancer team available in SUS from January 2018 to January 2020 (before the COVID-19 pandemic).

[ ] Nurse

[ ] Psychologist

[ ] Nutritionist

[ ] Physiotherapist

[ ] Pharmacist

[ ] Other ________________

16. Now the question is regarding the availability of palliative care services for your metastatic breast cancer patients treated via SUS. Evaluate the following sentence:

In SUS, I have a specialized palliative care service available to manage my metastatic breast cancer patients.

[ ] Strongly agree

[ ] Agree

[ ] Neither agree nor disagree

[ ] Disagree

[ ] Strongly disagree

(If Strongly agree or Agree)

16.b. You have responded that you strongly/agree to have a specialized palliative care service available to manage your metastatic breast cancer patients treated via SUS. Choose the options that best describe that service:

[ ] the palliative care service is offered to SUS patients at the same institution that they receive cancer treatment

[ ] the palliative care service is offered to SUS patients in an institution that is different from the institution they receive cancer treatment

[ ] Other. Specify: ________________________

17. The next question is about breast cancer patients treated via SUS who die at home. Evaluate the following statement:

At the service you work for in SUS, there is an established administrative procedure to generate death certificates for those breast cancer patients who died at home.

[ ] Strongly agree

[ ] Agree

[ ] Neither agree nor disagree

[ ] Disagree

[ ] Strongly disagree

18. Now the question focuses on the medications that are prescribed and given (dispensed) to breast cancer patients in SUS. Evaluate the following statement:

A dispensing sector provides my breast cancer patients with supportive drugs, such as anti-emetics and analgesics, at the institution I work for in SUS.

[ ] Strongly agree

[ ] Agree

[ ] Neither agree nor disagree

[ ] Disagree

[ ] Strongly disagree

19. This question is about whether your patients treated via SUS continue having medical visits with oncologists after they have been prescribed palliative care alone.

Evaluate the following sentence:

My breast cancer patients treated via SUS who had their chemotherapy interrupted due to metastasis with previous treatments and loss of performance status continue having medical visits with an oncologist after they have been prescribed palliative care alone.

[ ] Strongly agree

[ ] Agree

[ ] Neither agree nor disagree

[ ] Disagree

[ ] Strongly disagree

(If strongly agree or agree)

19.b. You have responded that you strongly agree/agree that your patients treated via SUS continue having medical visits with an oncologist after they have been prescribed palliative care alone. Now the question is about the frequency of medical visits of your patients receiving palliative care via SUS.

Consider the same population of patients:

- Breast cancer patients
- Treated via SUS
- Chemotherapy interrupted due to metastasis with previous treatments and loss of performance status
- Receiving palliative care alone via SUS.

Based on your experience in SUS from January 2018 to January 2020 (before the COVID-19 pandemic), you saw those patients in SUS, on average:

[ ] Once every 30 days
[ ] Once every 60 days
[ ] At irregular intervals
[ ] I don't know
[ ] Other ________________

PRIVATE HEALTH SECTOR

1. Now the question is about the "typical" time from the diagnosis of metastatic breast cancer to the surgical treatment at the private institution you work for.

Consider you have a female patient who has been recently diagnosed with metastatic breast cancer. The patient is 54 years old and has never been treated for breast cancer before. Based on the careful evaluation of her clinical and pathological characteristics, it was concluded that she has an indication for surgical treatment. Considering the period between January 2018 and January 2020 (before the COVID-19 pandemic), what would be the waiting time (in days) from diagnosis to surgical treatment for that patient at the private institution you work for?

(Provide your best guess in days; any number from 1 to 1,000).

___________________ days

2. Consider a patient with metastatic breast cancer when she was first diagnosed with breast cancer ("de novo metastatic"). The patient has an indication to start treatment at the clinical oncology sector via a private institution.

Considering the period between January 2018 and January 2020 (before the COVID-19 pandemic), what would be the waiting time (in days) from diagnosis to clinical treatment for that patient at the private institution you work for?

(Provide your best guess in days; any number from 1 to 1,000).

__________________ days

3. This question is about how you treat your breast cancer patients in the private sector. Specifically, your preference regarding the first-line treatment for your metastatic breast cancer patients treated in the private sector.

Consider your hormone-positive/HER2- metastatic breast cancer patients treated in the private sector. Which first-line breast cancer therapy do you prefer to treat them?

[ ] Hormone therapy

[ ] Hormone therapy associated with a cyclin-dependent kinase inhibitor

[ ] oral Single-agent chemotherapy

[ ] venous single agent chemotherapy

[ ] Chemotherapy with a combination of agents (oral or venous)

[ ] Other ______________________

4. This question is again specifically focused on your hormone-positive/HER2- metastatic breast cancer patients treated in the private sector.

Having that population in mind, evaluate the following sentence:

It is easy to obtain approval from private health insurance companies to treat my patients with cyclin-dependent kinase inhibitors. Consider scenario after cyclin-dependent kinase inhibitors inclusion by Agencia Nacional de Saúde [ANS] in march 2021] Strongly agree

[ ] Agree

[ ] Neither agree nor disagree

[ ] Disagree

[ ] Strongly disagree

(if Disagree or Strongly disagree)

4b. You answered that it might not be easy to obtain approval from health insurance companies to treat your patients with cyclin-dependent kinase inhibitors in the private sector.

Based on your experience as oncologist in the private sector, difficulties in obtaining authorization for cyclin-dependent kinase inhibitors in the private sector happen:

[ ] with a single health insurance company only

[ ] with a few health insurance companies

[ ] with most health insurance companies

[ ] with all health insurance companies

[ ] Other. Please, specify: ________________________________

5. Now, the question is about what is your strategy when private health insurance companies deny treatment with cyclin-dependent kinase inhibitors in the private sector.

Consider hormone-positive/HER2- metastatic breast cancer patients treated in the private sector who need treatment with cyclin-dependent kinase inhibitors. However, the private health insurance company did not approve that type of treatment. Consider scenario after cyclin-dependent kinase inhibitors inclusion by Agencia Nacional de Saúde [ANS] in march 2021

For those cases in the private health sector, what would you do?

[ ] You would instruct patients to contact the health insurance company to appeal the decision (for example, patients try a second time to get approval). If the second attempt to get treatment with cyclin-dependent kinase inhibitors fails, you change the type of treatment.

[ ] You would instruct patients to obtain the medication via the legal system (e.g., judicialization).

[ ] You would not instruct patients to contact the health insurance company and, as a result, would change the treatment to hormone therapy alone.

[ ] You would not instruct patients to contact the health insurance company and, as a result, would change the treatment to chemotherapy.

[ ] Other. Please, specify: ____

6. This question focuses on the probability of changing the health insurance company's decision without using the legal system (i.e., without judicialization).

Consider a hormone-positive/HER2- metastatic breast cancer patient treated in the private sector who needs treatment with cyclin-dependent kinase inhibitors. The private health insurance company did not approve that type of treatment.

Based on your experience in the private health system after march 2021 (after cyclin-dependent-kinase inhibitors inclusion by Agência Nacional de Saúde [ANS] when patients contact the health insurance company to revert the initial unfavorable decision, what was the overall chance of reverting that decision? (e.g., the treatment with cyclin-dependent kinase inhibitors was initially denied, but during the second attempt, the health insurance company decided to pay for it)

(answer in percentage, any value between 0 to 100%)

____

7. Now the question is whether you receive more money (higher payments) when specific treatments are prescribed in the private health sector.

Consider your hormone-positive/HER2- metastatic breast cancer patients treated in the private sector who need treatment with cyclin-dependent kinase inhibitors.

Evaluate the following sentence:

I receive more money (e.g., I am paid more) when I prescribe a treatment involving chemotherapy than when I prescribe hormone therapy combined with cyclin dependent kinase inhibitors

[ ] Strongly agree

[ ] Agree

[ ] Neither agree nor disagree

[ ] Disagree

[ ] Strongly disagree

(if Strongly agree or Agree)

8. You agreed that you receive more money when you prescribe a treatment involving chemotherapy than when you prescribe hormone therapy combined with cyclin dependent kinase

inhibitors.onsidering your experience as an oncologist in the private sector between January 2018 and January 2020 (before the COVID-19 pandemic), evaluate the following sentence:

The higher payments influence the therapeutic strategy I choose for my patients.

[ ] Strongly agree

[ ] Agree

[ ] Neither agree nor disagree

[ ] Disagree

[ ] Strongly disagree

9. The next question is whether the risk of QT interval prolongation reduces the chances of prescribing ribociclib.

According to recent studies, ribociclib can prolong the QT interval in hormone-positive/HER2- metastatic breast cancer patients.

Considering your experience as an oncologist in the private sector between January 2018 and January 2020 (before the COVID-19 pandemic), evaluate the following sentence:

I am less inclined to prescribe ribociclib to my breast cancer patients because of the risk of QT interval prolongation

[ ] Strongly agree

[ ] Agree

[ ] Neither agree nor disagree

[ ] Disagree

[ ] Strongly disagree

10. Evaluate the following sentence:

Between January 2018 and January 2020 (before the COVID-19 pandemic), in the private health sector, I had support from a multidisciplinary cancer team to manage my metastatic breast cancer patients at the private service I work for.

[ ] Strongly agree

[ ] Agree

[ ] Neither agree nor disagree

[ ] Disagree

[ ] Strongly disagree

(If Strongly agree or Agree)

10.b. This question is regarding the characteristics of the multidisciplinary cancer team that supported the management of your metastatic breast cancer in the private health sector. Select all professional categories that were part of the multidisciplinary cancer team available at the private institution you worked for from January 2018 to January 2020 (before the COVID-19 pandemic).

[ ] Nurse

[ ] Psychologist

[ ] Nutritionist

[ ] Physiotherapist

[ ] Pharmacist

[ ] Other ________________

11. Now the question is regarding the availability of palliative care services for your metastatic breast cancer patients treated via the private health sector. Evaluate the following sentence:

In the private health sector, I have a specialized palliative care service available to manage my metastatic breast cancer patients.

[ ] Strongly agree

[ ] Agree

[ ] Neither agree nor disagree

[ ] Disagree

[ ] Strongly disagree

(If Strongly agree or Agree)

11.b. You have responded that you strongly/agree to have a specialized palliative care service available to manage your metastatic breast cancer patients treated via the private health sector. Choose the options that best describe that service:

[ ] the palliative care service is offered to my private patients at the same private health institution that they receive cancer treatment

[ ] the palliative care service is offered to my private patients in an institution that is different from the institution they receive cancer treatment

[ ] Other. Specify: ________________________

12. This question is about whether your patients treated via the private health sector continue having medical visits with oncologists after they have been prescribed palliative care alone.

Evaluate the following sentence:

My breast cancer patients treated via the private health sector who had their chemotherapy interrupted due to metastasis with previous treatments and loss of performance status continue having medical visits with an oncologist after they have been prescribed palliative care alone.

[ ] Strongly agree

[ ] Agree

[ ] Neither agree nor disagree

[ ] Disagree

[ ] Strongly disagree

(If Strongly Agree or Agree)

12.b. You have responded that you strongly agree/agree that your patients treated via the private health sector continue having medical visits with you after they have been prescribed palliative care alone. Now the question is about the frequency of medical visits of your patients receiving palliative care via the private health sector.

Consider the same population of patients:

- Breast cancer patients
- Treated via the private health sector

- Chemotherapy interrupted due to metastasis with previous treatments and loss of performance status
- Receiving palliative care alone via the private health sector .

Based on your experience in the private health sector from January 2018 to January 2020 (before the COVID-19 pandemic), you saw those private patients, on average:

[ ] Once every 30 days

[ ] Once every 60 days

[ ] At irregular intervals

[ ] I don't know

[ ] Other ________________
